# Potential chemical risks from tattoos and their relevance to military health policy in the United States

**DOI:** 10.1057/s41271-023-00403-y

**Published:** 2023-03-13

**Authors:** James D. Blando, Blas A. Guigni

**Affiliations:** 1 School of Community and Environmental Health, Old Dominion University, 4608 Hampton Blvd, Norfolk, VA 23529 USA; 2 Toxicology Directorate, Army Public Health Center, Aberdeen Proving Ground, MD 21010 USA

**Keywords:** Tattoo, Disability payments, Skin, Veterans, Military, Health policy

## Abstract

We summarize and consolidate disparate sources of information about the practice of tattooing and its potential implications for military population health and policy. Each branch of the United States military has policies about tattoos for service members, but these have varied over time and do not cover health protection. The number of veterans receiving disability payments and the cost of those payments has been rising over time; the broad category of skin conditions accounts for 11% of disability claims. Any additional factor, such as tattoos that may increase the occurrence of adverse skin reactions, can substantially impact veteran benefit expenses and budgets. This may be a consideration for the military as it evaluates its policies related to tattoos among service members.

## Key messages

- The popularity of tattoos has dramatically increased in recent years and thus it is important to understand their health risks and potential impact on healthcare costs.
- The practice of tattooing can result in both infectious disease risks and chemical toxicity risks.
- A better understanding of the potential impact of tattoo risks on military disability payments for skin conditions may be worthwhile given the popularity of tattoos among military personnel.

## Introduction

This narrative review summarizes and consolidates disparate sources of information about the practice of tattooing and its potential implications for military policy. Our main purpose in conducting this review was to identify challenges to military policy formulation, summarize what is known and highlight information gaps, and make recommendations for future research.

Tattoos have become increasingly popular, especially in the United States. Each branch of the United States military has policies about the appearance and placement of tattoos for service members (Army: AR 670-1, Navy: NAVPERS 15665J, Air Force: AFI 36-2903, Marine Corps: MCO 1020.34H, Coast Guard: COMDTINST M1020.6K). These policies have varied over time and do not cover health protection. Currently, the United States Department of Defense allows tattoo vendors on military bases. Although the US Code of Federal Regulations (21 CFR Parts 73, 74, and 82) lists approved color additives for topical consumer cosmetic use, and despite the prevalence of tattoos, the United States Food and Drug Administration (FDA) has not approved any color additives for injection into the skin [1]. Currently, the FDA does not direct substantial resources to tattoo product evaluations and safety assessments, leaving these products mostly unregulated in the United States [2]. This may explain why the European market surveillance system RAPEX found that tattoo inks with the highest number of risk alerts were from US manufacturers [1, 3].

In the European Union (EU), there has been substantially more regulation and evaluation of tattoo products over the last 20 years than previously. The Council of Europe published resolutions (ResAP(2003)2) [4] and a revision (ResAP(2008)1) [5] to establish a framework for national laws addressing risks of tattoo products and practices. This framework included lists of banned substances for tattoo inks, typically compounds that were carcinogenic, mutagenic, or showed reproductive toxicity. The framework, however, is not legally binding and not all EU countries have endorsed or adopted these recommendations; only 10 countries base their national legislation on the resolutions [6]. The General Product Safety Directive (GPSD) (Directive 2001/95/EC), CLP (Classification, Labeling, and Packaging) (EC No 1272/2008), and REACH (EC No 1907/2006) regulations cover tattoo products in EU countries that have enacted no specific law [1, 6]. The variability in requirements across the EU may have contributed to products on the market in the EU that do not meet the requirements of the ResAP(2003)2 and the revised ResAP(2008)1 guidelines. The variability has encouraged proposal of further chemical use restrictions under REACH [6]. The Danish Environmental Protection Agency has issued a comprehensive set of guides to help improve compliance with new REACH requirements. These cover tattoo inks and provide extensive background on activities in the EU that address tattoo ink hazards [7]. These recommendations are consistent with themes published previously by the Danish Environmental Protection Agency, such as:

- needing safety risk assessments to be conducted on tattoo inks,
- banning the use of carcinogenic, mutagenic, reproductive toxins in inks, banning azo compounds that are known to degrade to carcinogenic amines,
- banning inks containing substantial levels of polycyclic aromatic hydrocarbons or lead,
- labeling the chemical content of inks to enhance consumer awareness,
- assuring inks are sterile and have an expiration date [7].

The variety and diversity of ink formulations contribute to the difficulty of evaluating the chemical toxicity and health risk of tattoos. It is unclear if consumers and general health professionals understand the potential risks of tattoos.

## Materials and methods

We carried out a literature search with PubMed and Google scholar using the following keywords or phrases: tattoo (ink) exposure, tattoo number per year, tattoo ink containers, tattoo colorants, tattoo toxicity, tattoo composition, tattoo pigments, tattoo dyes, tattoo ingredients, and tattoo ink market. This yielded 403 articles, most written in English; we translated three. The majority were not directly related to human physical health consequences. Instead, they dealt with the sociological, psychological, or other behavioral characteristics and motivations around tattoos and associated culture. Our inclusion criteria required articles to have been peer reviewed or published by a government agency, published after the year 2001, and to contain information relevant to human physical health risk. After review, a total of 53 articles remained for inclusion, most published within the last 10 years.

## Results

### Prevalence

The popularity of tattoos has increased over the years, particularly in the United States. A 2003 Harris poll found that 16% of US adults have tattoos. A 2015 Harris Poll reported that 29% of US adults are tattooed, including nearly half of Millennials (47%; those born between 1980 and 1999) and over a third of Gen Xers (36%; those born between 1960 and 1979). Among those with tattoos, seven in ten (69%) have two or more, with average total tattoo coverage at 400 cm^2^ [8]. Also, 19% of Americans without a tattoo consider getting one. Tattoos have become more socially acceptable. The demographics of individuals getting tattoos among the general public have expanded to include people in higher socioeconomic strata and older individuals [9]. A 2012 study of Australian citizens found the highest rates overall in men, but the highest rates by age group were women in their 20's [9, 10]. Kluger summarized studies showing that women have surpassed men in the United States [11]. This change may have implications for military service members and their families.

Only a few articles specifically addressed or collected data on military service members and their tattoos. A survey of 1897 Taiwanese service members found that roughly 25% had tattoos [12]. In a small convenience sample survey of US service members with tattoos (*n* = 122), roughly 57% of them, obtained their tattoos before enlisting. Presumably, the other 43% procured tattoos after enlisting; 7% of those did so during deployment [13]. A survey by Lande et al. also found that service members who got tattoos during deployment generally used local tattoo artists at their deployment location or had a fellow US service member administer the tattoo [13]. Kluger discussed use of unlicensed parlors in many places worldwide, citing an Italian example where 73% of high school students surveyed obtained body art at unlicensed facilities [11]. Lande et al*.* also found that female service members took tattoos at a slightly later age than men (23.2 years old vs. 19.6, respectively) but, on average, had the same number of tattoos as men [13]. Marines had more tattoos than members of the other armed service branches (average 5.8 tattoos versus roughly 3.2), higher-ranking officers had fewer than service members at the lower ranks, and the most common tattoo included the name of a person [13].

### Chemical composition of tattoo inks

Inks are highly variable in formulation and manufactured worldwide. Inks are comprised of pigments, carriers such as binding agents or solvents, additives such as surfactants and preservatives, and some combination of impurities and fillers [14]. Studies have shown that poor quality control and lack of standardized good manufacturing practices (GMP) at facilities have resulted in 10–20% of the inks sold containing pathogenic bacteria such as Staphylococcus, Clostridium, Streptococcus, and Pseudomonas, and might even contain fungal and viral pathogens [15, 16]. The purity of the inks used in tattoos is often less than 80%, meaning a substantial amount of impurities in ink [17]. It may be difficult to characterize any product due to a lack of regulatory oversight, lack of quality control during manufacturing, and a seemingly endless variety of formulations available from many and diverse sources.

Tattoos involve deposition of pigment particle aggregates into the skin’s dermis. Individual ink pigment particles exist at a range of sizes, from micrometer to nanoparticle sizes [18–21] and may contain both amorphous and crystalline particles including silicates [21]. The US Food and Drug Administration (FDA) developed a chemical library of pigments in tattoos, including inorganic compounds, organometallic compounds, monoazo and diazo compounds, indigoid compounds, and oxazine compounds [22].

Tattoos administered before the early 1980s often involved inorganic metallic pigments including oxides of iron and mercury [23]. Two studies reported a variety of metals, including oxides of titanium, cadmium, aluminum, chromium, cobalt, iron, zinc, and sulfides of mercury and cadmium in tattoo inks [24, 25]. Bocca et al*.* found aluminum, titanium, and copper particles in tattoo inks purchased in Italy; they also reported use of barium sulfate and magnesium oxides [20, 26]. Forte et al*.* found nickel, chromium, cadmium, and mercury-containing particles in tattoo inks [27]; Karbowska et al*.* found thallium ranging up to 0.43 µg/g in ink samples obtained from Israel and Italy [28], and Bocca et al*.* found hexavalent chromium impurity concentrations in tattoo inks ranging from roughly 0.2–4 µg/g [29]. Since about the mid-1980s, these metals are often substantial impurities in the ink formulation of tattoo inks. Schreiver et al*.*, have also suggested that needle wear may result in chromium and nickel particle deposition in the skin, mainly when the ink contains abrasive titanium dioxide particles [30].

Many black ink pigments contain carbon black and polycyclic aromatic hydrocarbons due to their production via combustion processes [31]. Lehner et al*.* found dibutyl phthalate, hexachloro-1,3-butadiene, metheneamine, dibenzofuran, benzophenone, and 9-fluorenone in black pigments used in tattoos [32]. In the early 1990s, the organic-based ink classes of azo compounds (diverse class with double-bonded nitrogen = nitrogen) used for many purposes have become standard components of tattoo pigments [23]. Some manufacturers have used sulfonated forms of azo compounds in foodstuffs because sulfonation increases excretion substantially and reduces toxicity [33]. Azo compounds popularity across many applications results from product performance, where the colorant needs to be resistant to fading and retain its original tone and shade. European regulations, however, have discouraged use of some azo compounds, considered by these regulators as potentially carcinogenic, and use of other complex polycyclic organic pigments commonly found in plastics, paint, and other coatings that have entered the tattoo market (phthalocyanines, quinacridones, diketopyrrolopyrroles, quinophthalones, triphendioxazines, perinone, anthraquinones, perylenes, and rhodamines) [34]. Schreiver et al*.* also found several polymers and thickener additives in popular tattoo inks, such as silicones, polyethylene glycol, polyvinylpyrrolidone, acrylates, polystyrene, and organic resin shellac [34]. Dirks summarized ink formulations and their pigments, solvents, thickeners, binders, preservatives, surface active additives, and fillers [14]. The toxicity of these complex organic ink product mixtures is poorly characterized. They may be associated with potential adverse health outcomes, such as carcinogenicity, irritation, or sensitization [34, 35].

### Tattoo ink toxicology

Little is known about the toxicology of tattoo inks and their distribution, metabolism, and excretion from the body [36]. Although a person may not perceive a fading or changing appearance of a tattoo, an animal study showed that 30% of the tattoo pigment may degrade within the skin after several weeks. Sunlight exposure can increase this degradation substantially [37, 38] due to the decomposition of larger pigment particles by UV radiation. There remains some debate about the ability of tattoo inks to disperse throughout the body and interact with tissues and organs in the body [16]. Clinicians’ presentations of patients at clinics and reported in clinical case series have produced most of the awareness of adverse chemical reactions. There is one notable exception: several studies have detected the presence of pigment particle aggregates in lymph nodes and blood vessels near the site of tattoo application. The translocation of pigments to the lymph nodes suggests that the particles can migrate over time and that smaller particle aggregates appear to migrate more readily [39, 40]. Researchers have shown this in animal studies [41] and human studies [19, 30, 39, 40, 42]. Schreiver et al*.* found translocation of pigment particles to lymph nodes in human postmortem samples [30]. Pigment particles can result in a blockage of lymphatic fluid flow, causing lymphedema [15, 43]. It remains unknown if pigment particles, which can be in nanoparticle form, translocate to other areas or organs of the body, as shown for other types of nanoparticles [44]. No studies yet show if there are implications for the fetus of pregnant women.

A few studies have shown that tattoo inks may be associated with molecular free radical production [31], oxidative stress [45], and other less well-defined enzymatic processes [16]. Bil et al*.* showed that tattoo inks have the distinct potential to cause keratinocytes to release IL-18 and act as both irritant and sensitizer in a model in vitro test system [46]. Organic pigments used in tattoo inks typically contain azo bonds or other organic cyclic compounds containing nitrogen. These compounds can undergo degradation and form carcinogenic aromatic amines [16]. Agnello and Fontana analyzed inks for aromatic amines and found that 40% of the inks they tested contained carcinogenic aromatic amine compounds [47]. They found these carcinogens commonly in red and yellow colors or derivatives (orange). The most common aromatic amines detected were o-toluidine and o-anisidine, likely the result of reductive cleavage of the azo bond. Many of these inks (44% of those positive for aromatic amines) contained relatively large quantities, with over 100 mg of aromatic amine per kilogram of ink [47]. The degradation of the pigment compounds to toxic by-products can be induced by exposure to sunlight [35, 41, 48, 49], laser light during tattoo removal [34, 35, 49], and potentially from biochemical reactions in the body forming various haptens [15, 50]. Some researchers have speculated about an association of tattooing with skin cancers [34, 35, 48]. Several case reports involved melanomas in the site of tattoos, but the causal link remains uncertain, and could be coincidental [35, 48].

Cui et al*.* found that a common yellow pigment used in tattoos could photo-decompose into metabolites that are imine compounds [23]. Hauri and Hohl reported that phthalocyanins, quinacridones, and dioxazines were stable in vitro, but the azo pigments readily degraded in simulated sunlight and laser light [49]. Photo-degradation has been intuitively known among tattoo enthusiasts even though both the inorganic and organic pigments are relatively resistant to fading. Aware consumers have recognized that tattoos should be protected from the sun. Fifty-two percent of complaints about adverse tattoo reactions among a convenience sample of beachgoers related to sun exposure of their tattoos [51]. Other mechanisms have also been implicated with tattoo pigment degradation. Sun et al*.* found that *Staphylococcus aureus* bacteria, a common microbe on human skin, can reduce some azo compounds used in cosmetics to aromatic amines [52].

Delayed allergic reactions may result from the slow degradation of the tattoo over time and likely result from haptens forming with degradation products from the tattoo [15, 50]. Researchers have noted, in human case series, that sensitization is an important adverse reaction to tattoos. Bil et al*.* described the adverse outcome pathway (AOP) for skin sensitization resulting from degradation of tattoo inks, where the degradation products form haptens by attaching to skin proteins (initiating event), followed an immunogenic response with release of IL-18 and TNF-α causing sensitization, activation of antigen-presenting immune cells (such as Langerhans cells), followed by activation of T cells and subsequent immune response [46]. This immune response results in the clinical presentation and symptoms of patients as an adverse allergic reaction to a tattoo. These adverse reactions can be delayed from weeks to years after administration of a tattoo because it is a sensitization reaction from tattoo ink degradation products. This degradation process has highly variable time frames among individual patients.

### Case reports of injuries

Our literature search did not yield any comprehensive epidemiologic studies that evaluated and determined incidence or prevalence of adverse reactions to tattoo procedures and products. Two studies described surveys that found that 6% of people in Germany who received tattoos reported persistent skin problems such as edema or itching [53]. Another survey found that one month after receiving a tattoo, 3.8% of American adults still experienced pain, and 21.2% still experienced itching [54]. These types of symptoms related to adverse events associated with tattoos have been shown to impact quality of life substantially and are associated with anxiety, depression, and sleep problems [55].

The largest source of information about the chemical risk of tattoos and their associated compounds is from clinical case reports. Generalization of findings is limited because they do not involve scientific study designs. They are extremely valuable, however, because they report on ‘real-world’ experiences among patients presenting with apparent effects from tattoos. These clinical reports have estimated that 33% of those with tattoos report having some adverse reaction over time, typically involving itching, swelling, crusting, or other relatively minor dermatological conditions [15, 43]. A small percentage of patients report more serious reactions including substantial hyperkeratosis, ulcerated tissue that can become necrotic, and lymphatic pathologies such as lymphedema [43, 56, 57]. Some case reports include tattoo reactions co-occurring with sarcoidosis [58]. Patients sometimes opt for removal of a tattoo due to the potential for adverse reactions to it [59]. Unfortunately, laser removal can release more tattoo ink degradation products and smaller aggregate pigment particle sizes [60], causing mild or moderate allergic reactions [61]. Clinicians may view the longer-term benefits of removal to outweigh these shorter-term risks. The medical literature contains many case series, frequently with red inks [41, 50, 57, 62], that suggest tattoos can cause adverse allergic reactions, especially dermatologic reactions. Several research studies have reported sensitization reactions to ink ingredients, such as paraphenylenediamine, with black Henna tattoos [63–65]. These do not involve an injection, just a surface application of ink. Frequently, sensitization reactions appear after a delay. Some patients have reported reactions within weeks after getting tattoos; others reported complaints from months to years later [15, 43]. Usually, the reactions are localized at the tattoo site [66], but several case reports in the series showed regional, distal, or potentially systemic sensitization reactions [15, 43]. One case report associated a reaction of a patient during laser removal of one tattoo on her wrist with an inflammatory response at other tattoo sites in other areas of the body where there was no laser treatment [67]. This observed reaction caused the reporter to hypothesize that allergens released during laser treatment caused a systemic immune reaction to all body areas containing tattoo pigments [67]. In each of these case reports, the dermatologists ruled out infectious or other causes of these adverse skin reactions and therefore strongly suspected involvement of the tattoo itself.

The toxicology information and these case reports suggest that tattoos may be hazardous to the general population—important given increases in popularity and numbers of tattoos in humans worldwide. This information is important for consumers, health care providers, and tattoo artists who need to be keenly aware of some contraindications to tattoos, such as for persons with chronic skin disorders (such as eczema), diabetes (due to potential delayed wound healing), heart disease (due to potential infective endocarditis), blood disorders (such as hemophilia), individuals who are immunosuppressed, and individuals with immune system dysfunction or autoimmunity [68].

## Discussion

### Implications and policy issues

The United States Food and Drug Administration (USFDA) regulates tattoo inks as cosmetics but does not put resources into the evaluation of tattoo inks and pigments. Thus, no one performs pre-market review or approval of tattoo inks and the United States (US) is essentially without regulation of what components may go into tattoo inks. In Europe, tattoo legislation is much more extensive. Some regulations cover the EU, and others are country specific. Experiences in Europe point out several key challenges with conducting risk assessments for tattoo inks. The European Commission JRC report Work Package 1 [69] and Work Package 3 summarize these [70]. European efforts help to identify needs for: standardized uniform guidelines on risk assessment practices, development of standardized and validated analytical chemistry methods for the analysis of inks, data on ink degradation products as they breakdown over time, better understanding of the biological behavior and toxicokinetic properties (ADME) of inks and pigments when applied to human skin, guidelines on proper adverse endpoint selection in toxicology studies so that appropriate NOAEL and LOAEL values can be determined, and the need for large-scale epidemiological studies with enough statistical power to detect effects [69]. Further complicating development of effective regulatory policies is lack of an appropriate model for evaluating the toxicological effects of tattoo pigments injected into the dermis and their long-term consequences. These challenges plus the multitude of products available for purchase worldwide present opportunities for further investigation and development of effective risk assessment and management strategies. Variability in policy and legislation around the world makes it difficult to harmonize risk management approaches. Ink products representing elevated risk can be difficult to remove from the marketplace because, for example, products banned in certain countries in Europe may still be sold in the United States and, hence, people around the world may be able to purchase them on-line.

Non-infectious reactions recorded in the literature could impact warfighter readiness and health across the range of adverse reactions from mild through more severe outcomes. Wenzel et al*.* [53] showed that 6% of people with tattoos experienced persistent skin problems. Carlsen and Serup [55] showed that 48% of people reporting adverse reactions to a tattoo also reported sleep problems and difficulty concentrating. Such effects could contribute to accidents among military personnel where sleep deprivation and lack of concentration are serious hazards, as in military aviation. Although no one has made accurate estimates of the incidence of more serious conditions, it is reasonable to assume that the more serious reactions detailed in case reports, such as sarcoidosis or lymphedema, would result in the removal of military personnel from duty or in imposition of restricted duty. Impacts on family, including spouses, could also distract deployed personnel, making them less effective in their duties.

Another impact, a widespread common consequence of tattoos in this population, could be claims for disability by service members upon separation from the military. The Veterans Administration regularly evaluates veterans for disability claims related to skin ailments [71]. In 2006, the Veterans Administration granted 1 in 5 veterans from the Iraq and Afghanistan conflicts disability payments (over 100,000 veterans at the time) and veterans filed 14% of these disability claims for skin conditions [72]. In 2014, the Congressional Budget Office reported the Veterans Benefits Administration compensated about 3.5 million veterans at the cost of 54 billion dollars during the prior year in 2013 [73]. The Veteran Benefits Administration budgeted roughly $87 billion to pay for claims in 2019 for approximately 5 million participants in the program [74]. From 2000 to 2013, the number of veterans receiving disability payments rose by 55% and the typical disability payment increased by 60%, consistent with the increasing number and severity of compensated injuries [73]. In 2013, the broad category of skin conditions accounted for 11% of disability claims [73]. Any additional factor, such as tattoos that may increase the occurrence of adverse skin reactions, may increase substantially veteran benefit expenses and budgets. These costs may be a consideration for the military as it evaluates its policies related to tattoos among service members.

## Conclusion

Many questions about tattoos remain unanswered. These arise from the complexity and inter-relationships among behavioral motivations, social benefits, military implications, and potential toxicological consequences. The challenge of conducting a thorough risk assessment of tattoo inks is difficult due to lack of standardized analytical and toxicology data, lack of adequate epidemiologic study, and variability in regulation and management approaches around the world. Many questions, such as the impact of ink mobilization and pregnancy risk, have not been studied. No one yet knows the relationship of tattooing to military-associated disability compensation expenses for skin conditions. The many questions present a worthwhile opportunity to better understand an increasingly common tattooing practice in the general population and military. Given emerging evidence that tattoos represent not only an infectious risk but also a chemical toxicity risk, further study is especially important. Efforts to support development of screening and detailed toxicology methods for the assessment of intra-dermally injected tattoo inks, comprehensive epidemiologic studies, and an impact assessment on military disability compensation for skin conditions would be extremely beneficial for risk assessment and management purposes.

## Data Availability

All articles and information related to this article may be obtained directly by contacting the corresponding author.
